# Ablation of dynamin-related protein 1 promotes diabetes-induced synaptic injury in the hippocampus

**DOI:** 10.1038/s41419-021-03723-7

**Published:** 2021-05-05

**Authors:** Gyeongah Park, Jong Youl Lee, Hye Min Han, Hyeong Seok An, Zhen Jin, Eun Ae Jeong, Kyung Eun Kim, Hyun Joo Shin, Jaewoong Lee, Dawon Kang, Hyun Joon Kim, Yong Chul Bae, Gu Seob Roh

**Affiliations:** 1 Department of Anatomy and Convergence Medical Science, Institute of Health Sciences, College of Medicine, Gyeongsang National University, Jinju, Gyeongnam 52727 Republic of Korea; 2 Bio Anti-Aging Medical Research Center, College of Medicine, Gyeongsang National University, Jinju, Gyeongnam 52727 Republic of Korea; 3 Department of Anatomy and Neurobiology, University of Tennessee Health Science Center, Memphis, TN 38163 USA; 4 Department of Anatomy and Neurobiology, School of Dentistry, Kyungpook National University, Daegu, 41944 South Korea; 5 Department of Physiology, College of Medicine, Institute of Health Sciences, Gyeongsang National University, Jinju, Gyeongnam 52727 Republic of Korea

**Keywords:** Neurodegeneration, Neurodegeneration, Diabetes complications

## Abstract

Dynamin-related protein 1 (Drp1)-mediated mitochondrial dysfunction is associated with synaptic injury in the diabetic brain. However, the dysfunctional mitochondria by Drp1 deletion in the diabetic brain are poorly understood. Here, we investigated the effects of neuron-specific Drp1 deletion on synaptic damage and mitophagy in the hippocampus of a high-fat diet (HFD)/streptozotocin (STZ)-induced diabetic mice. HFD/STZ-induced diabetic mice exhibited metabolic disturbances and synaptic damages. Floxed *Drp1* mice were crossed with Ca^2+^/calmodulin-dependent protein kinase IIα (*CaMKIIα*)-*Cre* mice, to generate neuron-specific *Drp1* knockout (*Drp1cKO*) mice, which showed marked mitochondrial swelling and dendritic spine loss in hippocampal neurons. In particular, diabetic *Drp1cKO* mice exhibited an increase in dendritic spine loss and higher levels of oxidative stress and neuroinflammation compared with diabetic wild-type (WT) mice. Diabetic WT mice generally displayed increased Drp1-induced small mitochondrial morphology in hippocampal neurons, but large mitochondria were prominently observed in diabetic *Drp1cKO* mice. The levels of microtubule-associated protein 1 light-chain 3 and lysosomal-associated membrane protein 1 proteins were significantly increased in the hippocampus of diabetic *Drp1cKO* mice compared with diabetic WT mice. The inhibition of Drp1 adversely promotes synaptic injury and neurodegeneration in the diabetic brain. The findings suggest that the exploratory mechanisms behind Drp1-mediated mitochondrial dysfunction could provide a possible therapeutic target for diabetic brain complications.

## Introduction

Type 2 diabetes is a chronic disease that is characterized by low-grade chronic inflammation and insulin resistance, which result in hippocampal vulnerability and synaptic damage^1,2^. The hippocampus is particularly susceptible to oxidative stress and inflammation^3^. Diabetes adversely affects the brain and confers a major risk for Alzheimer’s disease (AD)^4,5^. Many studies have reported that the diabetic brain exhibits neuroinflammation, synaptic injury, mitochondrial dysfunction, and defective autophagy^6–8^.

Mitochondrial dynamics are regulated by fission and fusion^9^. Dynamin-related protein 1 (Drp1) is a mitochondrial fission protein that induces mitochondrial division on the outer mitochondria membrane by a guanosine triphosphate-dependent conformational change^10^. An increase in Drp1 protein expression is associated with pathophysiological events, such as diabetes, obesity, and neurodegenerative diseases^9,11–14^. For example, high-glucose conditions can cause excessive mitochondrial fission and result in the increased production of reactive oxygen species (ROS) via mitochondrial hyperpolarization, which produces several fragmented mitochondria^15–17^. According to several studies, excessive Drp1 expression can accelerate neurodegeneration due to increased interactions with phosphorylated Tau^18–20^. In contrast to increased Drp1 expression, Drp1 inhibition by mitochondrial division inhibitor 1 attenuates 1-methyl-4-phenyl-1,2,3,6-tetrahydropyridine-induced neurotoxicity and myocardial ischemia-reperfusion injury in diabetic heart^21,22^. It has been supported that the modulation of Drp1 activity provides protective effects against synaptic dysfunction and attenuate mitochondrial ROS production in the hippocampus of diabetic or AD model animals^13,23^. These findings suggested that Drp1 may play an important role in diabetes-induced synaptic dysfunction. Recent study has reported that neuron-specific *Drp1-*deficient mice have impaired synaptic transmission and reduced ATP levels^24^. It is unclear, however, whether synaptic damage caused by diabetes is exacerbated by Drp1 deficiency in hippocampal neurons.

Mitochondrial autophagy, also known as mitophagy, plays an important role in mitochondrial quality control and the removal of fragmented mitochondria^25^. Several studies have suggested that fission proteins are essential for mitophagy induction, and mitophagy is known to be involved in various neurodegenerative diseases^26,27^. However, whether mitophagy is involved in the effects associated with the neuron-specific deletion of Drp1 in the diabetic brain remains unknown.

In this study, to examine the consequences of blocked mitochondrial fission in diabetic neurons, *Drp1*^*lox*/lox^ mice were crossed with *CaMKII-cre-α* mice to generate neuron-specific *Drp1-*deficient (*Drp1cKO*) mice^24^. These mice exhibited neuronal-specific alterations in mitochondrial shape and distribution in the hippocampus. Here, we test whether Drp1 loss could aggravate diabetes-induced neurodegeneration in the diabetic brain. We sought to investigate the potential role of Drp1 in synaptic damage and mitophagy after diabetic induction. Our findings show that genetic loss of Drp1 can aggravate the diabetes-induced neuroinflammation, oxidative stress, and dendritic spine loss. In addition, we show that atypical large-sized autophagosomes are found to have large mitochondria in hippocampal neurons of diabetic *Drp1cKO* mice. These findings suggest that neuronal mitochondrial dynamics can play an important role in protection against synaptic damage, and that Drp1 may be a promising therapeutic target in neurodegenerative diseases for the treatment of synaptic damage.

## Materials and methods

### Animals

*Drp1*^*lox*/lox^ and *CaMKIIα-Cre* (6 weeks old) mice were received from Dr. Nakamura (University of California, San Francisco, USA); mitochondrial-targeted form of the fluorescent reporter Keima (mt-Keima) transgenic mice were obtained from Dr. Jeanho Yun (Dong-A University, Busan, South Korea); 3-week-old male wild-type (WT) (C57BL/6J) mice, 4-week-old WT (C57BL/6J^+/+^) and ob/ob (C57BL/6J^ob/ob^) mice, and 5-week-old male db/m and db/db (C57BL/6J^db/db^) mice were purchased from Central Laboratory Animal Inc (Seoul, South Korea). Animal studies were performed in accordance with the National Institutes of Health guidelines on the use of laboratory animals. The study protocol was approved by the Gyeongsang National University Animal Care Committee (GNU-160530-M0025). The mice were individually housed under a 12-h light/dark cycle.

### Genotyping polymerase chain reaction (PCR)

**Fig. 1:**
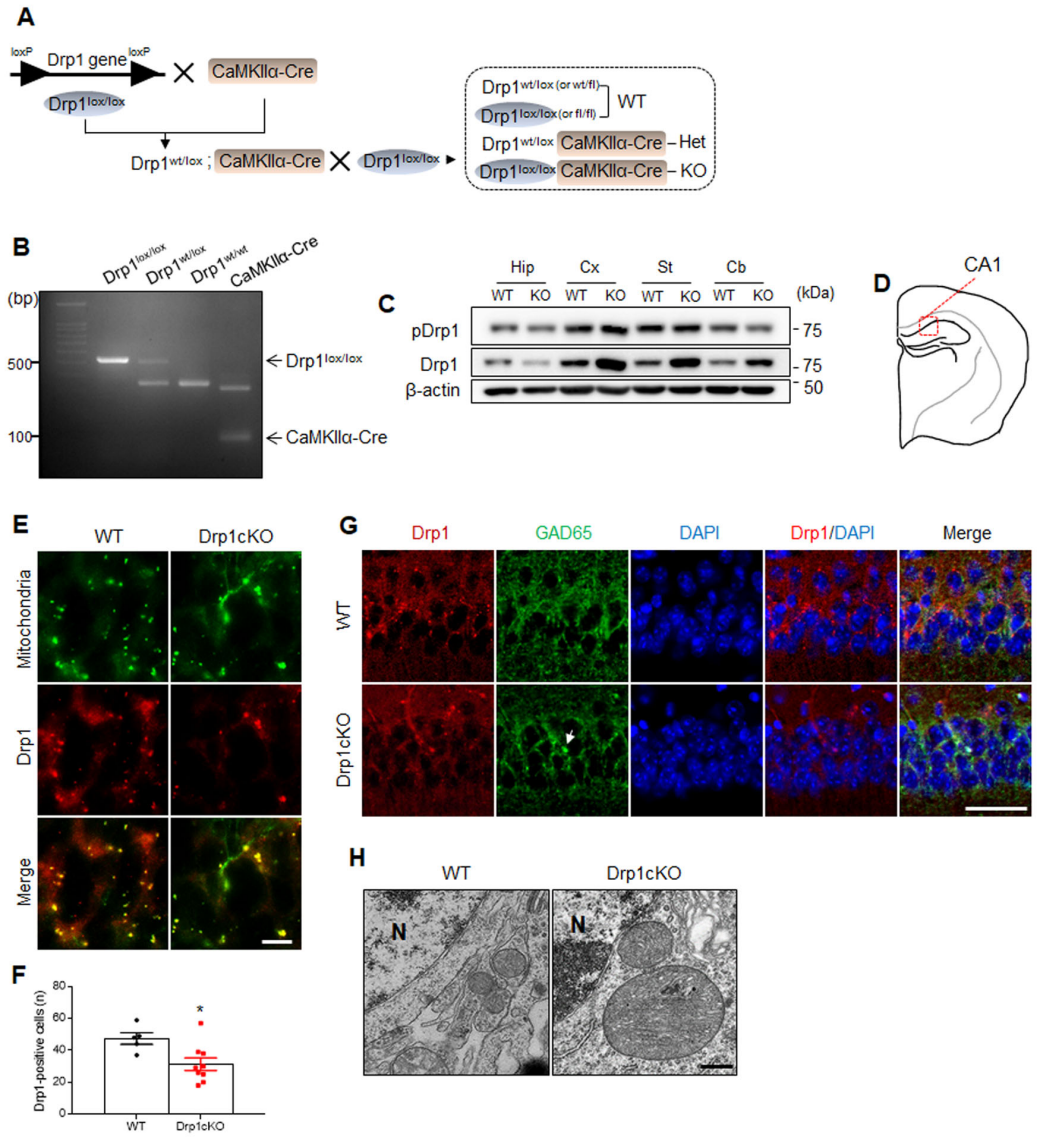
*Drp1cKO* mice exhibit alterations in neuronal mitochondrial morphology. **A** Breeding and *Drp1* knockout (KO) strategy using the Cre/loxP system. **B** PCR analysis was performed on *Drp1*^*lox/lox*^ and *CaMKIIα-Cre* mice. **C** Western blotting of pDrp1 and Drp1 levels in the hippocampus (Hip), crerebral cortex (Cx), striatum (St), and cerebellum (Cb). **D** Schematic coronal section illustrating the mouse brain in the hippocampal CA1 region. **E** Representative staining of Drp1 (red) and mitochondria (green) in the hippocampal CA1 region of wild-type (WT) and *CaMKIIα-Cre; Drp1*^*lox/lox*^ (*Drp1cKO*) mice. Scale bar = 5 µm. **F** The number of Drp1-positive neurons in the hippocampal CA1 region. **G** Representative staining of Drp1 (red) and glutamate decarboxylase 65 (GAD65, green) in the hippocampal CA1 region of WT and *Drp1cKO* mice. Nuclei were counterstained with DAPI (blue). Scale bar = 25 µm. White arrow indicates Drp1-positive GAD65 expression GABAergic neuron. **H** Representative electron microscopic images showing mitochondrial morphology in WT and *Drp1cKO* mice. Magnification 30,000×. Scale bar = 0.5 µm. N nucleus. Data are shown as the mean ± SEM. The indicated *p* values represent unpaired *t*-test in **F**. *p < 0.05 vs WT mice.

Genotyping PCR was performed using the primers listed in Fig. 1B and Supplementary Table S2. PCR reactions were conducted using the following cycle conditions: 2 min at 94 °C, followed by 35 cycles of 94 °C for 20 sec, 60 °C for 20 sec, and 72 °C for 20 min, and a final extension step at 72 °C for 2 min. Mt-Keima (Tg^+/+^) and WT (Tg^–^^/–^) mice were confirmed by PCR (Fig. 5C), using primers listed in Supplementary Table S2. Reactions were performed using the following cycle conditions: 5 min at 95 °C, followed by 30 cycles of 95 °C for 1 min, 61 °C for 1 min, and 72 °C for 1 min, and a final extension step at 72 °C for 7 min.

### Diabetic mouse models

Starting at 8 weeks of age, male mice were randomly divided into four groups (*n* = 20 per group). WT control (CTL) and *Drp1cKO* CTL mice were fed standard chow (normal diet [ND], 3.1 kcal/kg; Harlan Laboratories, Inc., Indianapolis, IN, USA) for 20 weeks (Supplementary Fig. S1A). The WT DM and *Drp1cKO* DM were fed a high-fat diet (HFD) (60 kcal% fat, 5.24 kcal/g; Research Diet, Inc., New Brunswick, NJ, USA) for 16 weeks. After 16 weeks, mice received a single dose of streptozotocin (STZ) (100 mg/kg), via intraperitoneal (i.p.) injection, and continued to be fed an HFD for 4 additional weeks. We used this mouse model to mimic type 2 diabetes, which is caused by impaired β-cell function and insulin resistance^28^. Similarly, mt-Keima (Tg^+/+^) mice were divided into two groups (*n* = 5 per group). ND-fed mt-Keima (mt-Keima CTL) mice were given ND for 20 weeks, and the mt-Keima diabetic model mice (mt-Keima DM) were fed an HFD for 16 weeks, given a single dose of STZ via i.p. injection, followed by the continued feeding with HFD for 4 more weeks. With respect to the HFD model, mice were divided into two groups and fed either HFD (*n* = 4) or ND (*n* = 3) for 20 weeks. WT, ob/ob, db/m, and db/db mice (*n* = 4 per group) were fed ND for 22 weeks. The mice had ad libitum access to food and water. Mice were weighed, and the fasting blood glucose was measured using an Accu-Chek glucometer (Roche Diagnostics GmbH, Mannheim, Germany).

### Glucose tolerance test (GTT) and insulin tolerance test (ITT)

GTT and ITT were performed, as previously described^29^. After overnight fasting, D-glucose (2 g/kg; Sigma-Aldrich) was intraperitoneally injected, and fasting blood glucose was measured before and after the injection, using an Accu-Chek glucometer (Roche Diagnostics GmbH). For ITT, after 6 h of fasting, ITT was performed on mice. Mice were injected with insulin (0.75 units/kg; Humulin-R; Eli Lilly and Company, Indianapolis, IN, USA), in 0.1 mL 0.9% normal saline. We measured blood glucose with an Accu-Chek glucometer.

### Measurement of metabolic parameters

After overnight fasting, mice (*n* = 8 per group) were anesthetized with zoletil (5 mg/kg; Virbac Laboratories, Carros, France), and the blood samples were collected and analyzed to measure serum glucose, total cholesterol, aspartate aminotransferase, and alanine aminotransferase levels, using enzymatic colorimetric assays from Green Cross Reference Laboratory (Yongin-si, South Korea). To measure insulin in serum, enzyme-linked immunosorbent assay (Shibayagi Co., Gunma, Japan) was performed, according to the manufacturer’s instructions.

### Tissue collection and histologic examination

Mice (*n* = 3 mice per group) were anesthetized with intramuscular zoletil (5 mg/kg) and perfused with 4% paraformaldehyde (PFA), in 0.1 M phosphate-buffered saline (PBS), to perform tissue analysis. After a 6 h fixation, mouse brains were immersed in 15, 20, and 30% sucrose, at 4 °C, until they sank. Brains were sliced into 30-μm sections. The liver, pancreas, and epididymal fat pad were processed for paraffin embedding and sliced into 5-μm sections. Deparaffinized livers and epididymal sections were stained with hematoxylin and eosin. To determine hepatic lipid accumulation, frozen liver sections (10 µm) were stained with Nile Red (Sigma-Aldrich, St. Louis, MO, USA) for 10 min and washed with PBS. The sections were visualized with a BX51 light microscope (Olympus, Tokyo, Japan), and digital images were captured and documented.

### Immunohistochemistry

Deparaffinized pancreatic sections (*n* = 3 mice per group) were placed in 0.1% H_2_O_2_ solution for 10 min, washed with PBS, and sections were treated for 30 min with diluted blocking serum. In a humidified container, slides were incubated with the insulin antibody (Supplementary Table S3), at 4 °C overnight, washed with PBS, and incubated with a secondary biotinylated antibody for 1 h, at room temperature. After washing, pancreatic sections were incubated in an avidin–biotin–peroxidase complex solution (Vector Laboratories, Burlingame, CA, USA) and developed with 0.05% diaminobenzidine (Sigma-Aldrich), containing 0.05% H_2_O_2_. The pancreatic sections were then dehydrated in graded alcohols, cleared in xylene, and mounted under a coverslip with Permount (Sigma-Aldrich). The immunostaining sections were visualized under a BX51 light microscope (Olympus).

### Immunofluorescence

For double-immunofluorescence staining, free-floating brain sections were incubated with primary antibodies (Supplementary Table S3), at 4 °C overnight, washed with PBS, and incubated with donkey secondary antibodies conjugated with Alexa Fluor 488 and 594 (Invitrogen, Carlsbad, USA). Nuclei were stained with 4′, 6-diamidino-2-phenylindole (DAPI, 1:10,000, Thermo Fisher Scientific, MA, USA). Immunofluorescence images of the sections were captured using a BX51-DSU microscope (Olympus, Tokyo, Japan). For the measurement of intensity of immunostained heme oxygenase-1 (HO-1) from brain sections, three fields (100 × 100 µm^2^) were randomly selected from each section (*n* = 3 per group).

### In vivo analysis of mitophagy using mt-Keima mice

Brain sections from mt-Keima mice were obtained, as described previously^30^. Briefly, mice brains were rapidly removed, washed with cold PBS, and cut into 1 mm coronal sections, containing the hippocampus. Brain sections were incubated for 1–2 min into PBS with DAPI. The sections were processed on the confocal dish (SPL, Pocheon, South Korea) and examined immediately with a confocal microscope (FV-1000, Olympus). Images were collected with filter sets for fluorescein isothiocyanate and a 594-nm excitation. DAPI images were collected with a 405-nm excitation. Calculation of mitophagy based on mt-Keima red signal was measured using ImageJ (National Institutes of Health, Bethesda, MD, USA, http://imageJ.nih.gov/ij). Four fields (20 × 20 µm^2^) in the confocal image were selected randomly. The average particles from mt-Keima CTL and mt-Keima DM were analyzed and normalized to the control level.

### Rapid Golgi staining

For rapid Golgi staining, mice (*n* = 3 mice per group) were i.p. anesthetized with zoletil (5 mg/kg), and brains were removed from the skull, as quickly as possible, and rinsed briefly in distilled water. Golgi staining was performed with an FD rapid Golgi Stain kit (PK 401; FD NeuroTechnologies, Inc., MD, USA), according to the manufacturer’s protocol. Coronal sections (150-µm) of Golgi-stained brains were cut with a vibratome (Leica VT1200S, Freiburg Germany). Slices were mounted on microscope slides, and sections were visualized under a BX51 light microscope (Olympus). Seven fields (20 × 20 µm^2^) were randomly selected to examine the spine number and density (*n* = 3 per group).

### Electron microscopy (EM)

EM was performed as previously described^31^. The mice (*n* = 3 in each group) were anesthetized with zoletil (5 mg/kg), perfused with saline, and fixed using both 4% PFA and 0.05% glutaraldehyde, in 0.1 M phosphate buffer (PB) (PH 7.4). The brains were fixed with the same fixative solution, for 2 h at 4 °C, and cut into 60-µm coronal sections using a vibratome. Then, the sections were rinsed with PB and osmicated in 1% osmium tetroxide (in PB) for 1 h. Sections were dehydrated in graded alcohols, flat-embedded in Durcupan ACM (Fluka, Buchs, Switzerland), between strips of Aclar plastic film (EMS, Hatfield, PA), and cured for 48 h at 56 °C. Small parts of the brain were cut out of wafers and placed onto blank blocks of resin. Thin sections were cut with a diamond knife, at 60 nm, and stained with uranyl acetate and lead citrate. Images, at 10,000× or 30,000× magnification, were obtained using an SC1000 CCD camera (Gatan, Pleasanton, CA), at an 80 kV accelerating voltage. The results were analyzed on light micrographs, taken from the 2–3 sections of the hippocampal CA1 region. Thirty micrographs, representing 592.12 µm^2^ each, from each mouse were obtained and used for quantification. To determine the mitochondrial number, mitochondrial size, autophagosome size, and autolysosome size, images were collected from the pyramidal cell layer of the CA1 region. Synapse and mitochondrial numbers in the presynaptic region were measured in the CA1 stratum radiatum. The morphological properties were quantified, and measurements made using ImageJ (National Institutes of Health).

### Western blot analysis

The hippocampi and other brain regions (cerebral cortex, striatum, and cerebellum) were dissected from mouse brains. Total lysis (*n* = 6–7 per group) was performed, as described previously, and homogenized using a lysis buffer^29^. α-tubulin and β-actin were used as controls to normalize total protein levels. To extract the mitochondria fraction (*n* = 3–4 per group), samples were homogenized using a mitochondrial isolation buffer (Thermo Fisher Scientific). Mitochondrial marker voltage-dependent anion channel 1 (VDAC1) was used as a control to normalize the levels of proteins in the mitochondrial fraction. The nuclear fraction (*n* = 3–4 per group) was separated by using a NE-TER^®^ Nuclear and Cytoplasmic Extraction kit (Pierce, Rockford, IL, USA), and nuclear maker p84 was used as a control to normalize protein levels in the nuclear fraction. Western blot analysis was performed, using standard methods. Proteins were immunoblotted with primary antibodies (Supplementary Table S3). Membranes were visualized using an enhanced chemiluminescence substrate (Pierce). Band densitometry was performed using the Multi-Gauge V 3.0 image analysis program (Fujifilm, Tokyo, Japan).

### Statistical analysis

PRISM was used to conduct statistical analysis (GraphPad Software Inc, San Diego, CA, USA). Group differences were determined by two-way analyses of variance, followed by a post hoc analysis with Tukey’s test. Student’s *t*-tests were used for HFD, ob/ob, and db/db models, to compare between two groups. All values are expressed as the mean ± standard error of the mean. A *p* value of <0.05 was considered significant.

## Results

### Drp1 loss causes alterations in mitochondrial morphology in hippocampal neurons

As published by other study group^24^, we bred floxed Drp1 mice with CaMKIIα (CaMKCre) mice. *Drp1*^*lox/lox*^ mice were crossed with *CaMKIIα-Cre* mice. *CaMKIIα-Cre; Drp1*^*lox/lox*^ (*Drp1cKO*) mice were generated by crossing heterozygous mice (Fig. 1A, B). In *Drp1cKO* mice, both phosphorylated (pDrp1) and total Drp1 expression levels were decreased in the hippocampus compared with those observed in WT mice, while other brain regions showed an increased total Drp1 protein in Drp1cKO mice (Fig. 1C). Immunofluorescent images revealed that *Drp1cKO* mice displayed a reduced number of Drp1-positive mitochondria compared with the WT mice (Fig. 1D–F). Furthermore, to characterize the deletion of Drp1 in *CaMKIIα*-expressing neurons in *Drp1cKO* mice, we performed double immunofluorescence with Drp1 and GAD65 antibodies (Fig. 1G). Compared to WT mice, a few Drp1-positive cells were co-localized with GAD65-positive GABAergic neurons in Drp1cKO mice. In particular, electron microscopic images showed large mitochondria in the area surrounding the nuclei of neurons of the hippocampal CA1 region of *Drp1cKO* mice compared with the WT mice (Fig. 1H). Immunofluorescence images revealed that *Drp1cKO* mice had mitochondrial marker or VDAC1-positive large perinuclear mitochondria, while *CaMKIIα* Cre recombinase had no effect on perinuclear mitochondria morphology (Supplementary Fig. S1). Taken together, these findings showed that *CaMKIIα*-expressing neuron-specific Drp1 depletion could cause prominent changes of mitochondrial morphology in hippocampal neurons.

### Effects of Drp1 deletion on synaptic damages and in the hippocampus of HFD/STZ-induced diabetic mice

Using our established diabetic mouse model (Supplementary Fig. S2A), HFD/STZ-induced diabetic phenotypes, including increased weight, insulin resistance, hepatic steatosis, adipose tissue macrophage infiltration, and hypercholesterolemia compared with the CTL mice (Supplementary Figs. S2B–S4). However, no metabolic differences were identified between the WT DM and *Drp1cKO* DM mice.

**Fig. 2:**
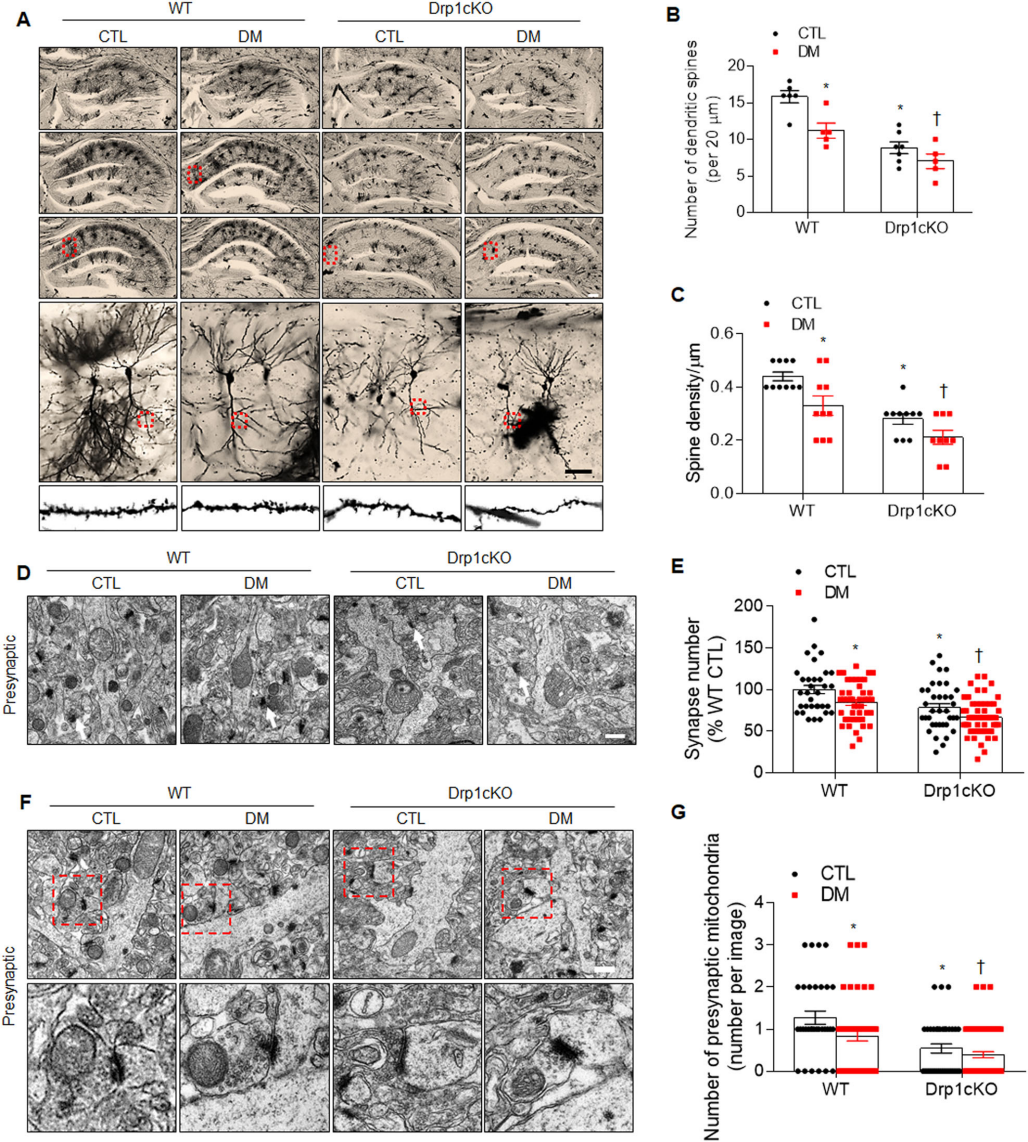
Effects of Drp1 deletion on synaptic damage in the hippocampus of HFD/STZ-treated mice. **A** Representative microphotographs of Golgi-stained hippocampus sections from WT and *Drp1cKO* mice, fed with either ND (CTL) or HFD/STZ (DM). Golgi-stained neurons were visualized in the hippocampal regions (*n* = 3 mice per group). High-magnification images (in lowest panels) from red box in upper panels. Scale bar = 200 µm. **B** Spine numbers and **C** spine density in hippocampal CA1 neurons. **D** Representative EM images showing the numbers of synapses in the longitudinal cross-section of an axon. Scale bar = 0.5 µm (*n* = 3 mice per group). White arrows indicate synapses. **E** Synapse counts in the hippocampus. **F** Representative EM images showing the number of mitochondria in the presynaptic region. High-magnification images (in lower panels) from red box in upper panels. Magnification 30,000×. Scale bar = 0.5 µm. **G** Measurements of mitochondrial numbers in the presynaptic region. Data are shown as the mean ± SEM. The indicated *p* values represent a two-way ANOVA followed by Tukey’s post hoc test. *p < 0.05 vs WT CTL; ^†^*p* < 0.05 vs WT DM.

Next, to determine whether Drp1 deletion affects synaptic damages, we performed Golgi staining to evaluate morphological changes in the dendritic spines of the hippocampal CA1 region. We found that the WT DM, *Drp1cKO* CTL, and *Drp1cKO* DM mice showed reduced dendritic branching and smaller dendritic spine trees compared with the WT CTL mice (Fig. 2A). In *Drp1cKO* DM mice, in particular, the dendritic spine numbers and dendritic spine density were lower than those in the WT DM mice (Fig. 2B, C). To further evaluate how Drp1 loss affects synaptic damage in the hippocampus, we performed EM. In the layer of the stratum radiatum of the hippocampus, *Drp1cKO* DM mice showed a reduced number of synapses compared with the WT DM or *Drp1cKO* CTL mice (Fig. 2D, E). In addition, we found that the number of presynaptic mitochondria was significantly decreased in both the *Drp1cKO* CTL and DM mice compared with that of the WT DM mice (Fig. 2F, G). The levels of pTau expression increased in the hippocampus of the WT DM, *Drp1cKO* CTL, and *Drp1cKO* DM mice compared with the level in the WT CTL mice (Supplementary Fig. S5A). In addition, immunofluorescence revealed fewer brain-derived neurotrophic factor-positive neurons in the hippocampal CA1 regions of the WT DM, *Drp1cKO* CTL, and *Drp1cKO* DM mice compared with the WT CTL mice (Supplementary Fig. S5B). These findings indicated that Drp1 deletion deteriorates diabetes-induced synaptic disruptions and reduction of presynaptic mitochondria in the diabetic brain.

### Effects of Drp1 deletion on oxidative stress and neuroinflammation in the hippocampus of HFD/STZ-induced diabetic mice

**Fig. 3:**
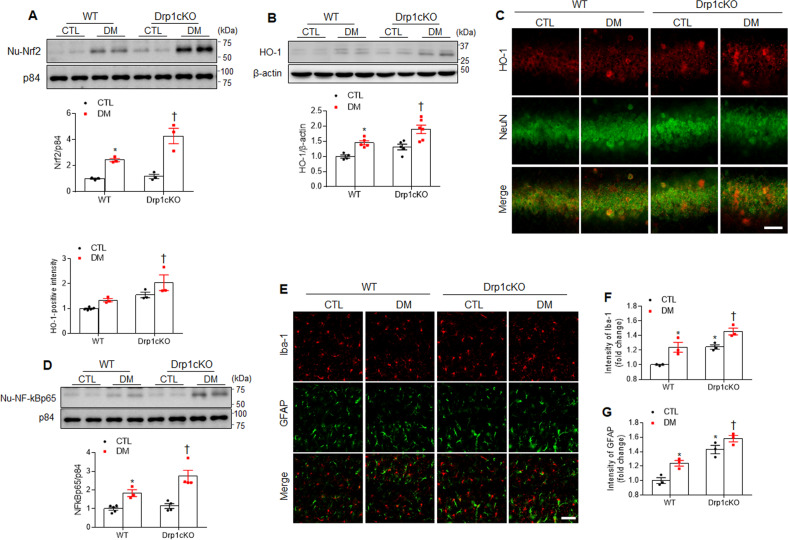
Effects of Drp1 deletion on oxidative stress and neuroinflammation in the hippocampus of HFD/STZ-induced diabetic mice. **A**, **B** Western blot and protein quantification analysis of nuclear (nu-) Nrf2 (**A**) and HO-1 (**B**) expression in the hippocampus (*n* = 3–4 mice per group). Densitometry values for Nrf2 and HO-1 were normalized against those for nuclear marker p84 and loading control marker β–actin, respectively. **C** Representative double immunofluorescence for HO-1 (red) and NeuN (green) (*n* = 3 mice per group). The intensity of HO-1 immunostained neurons in the hippocampal CA1 region were measured and presented as the fold change. Scale bar = 20 µm. **D** Western blot and protein quantification of nuclear (nu-) NF-κBp65 expression in the hippocampus (*n* = 3–4 mice per group). Densitometry value for NF-κBp65 was normalized against that for nuclear marker p84. **E** Representative images of immunofluorescence for Iba-1 (red) and GFAP (green) in the hippocampal CA1 regions. The intensity of Iba-1 **F** and GFAP **G** immunofluorescence. Scale bar = 20 µm. Data are shown as the mean ± SEM. The indicated *p* values represent a two-way ANOVA followed by Tukey’s post hoc test. *p < 0.05 vs WT CTL; ^†^*p* < 0.05 vs WT DM.

The nuclear factor erythroid-2-related factor 2 (Nrf2) and HO-1 protein levels were significantly higher in the *Drp1cKO* DM mice than in the WT DM mice (Fig. 3A, B). In particular, many HO-1-positive neurons were observed in the Drp1cKO DM mice compared to WT DM mice (Fig. 3C). As shown in Fig. 3D, Drp1 deletion increased the expression levels of nuclear factor-kB p65 in the *Drp1cKO* DM mice compared with the WT DM mice. Furthermore, double-immunofluorescence labeling revealed that diabetes or Drp1 loss led to an increased intensity in both the ionized calcium-binding adaptor molecule (Iba-1) and glial fibrillary acidic protein (GFAP), in the hippocampus (Fig. 3E–G). In particular, more high-intensity Iba-1-positive microglia and GFAP-positive astrocytes were observed in the *Drp1cKO* DM mice when compared to WT DM mice. Therefore, our data indicated that neuronal Drp1 loss adversely deteriorates oxidative stress and neuroinflammation in the diabetic hippocampus.

### Effects of Drp1 deletion on mitochondrial abnormality in the hippocampus of HFD/STZ-induced diabetic mice

Diabetic insult causes the increase of mitochondrial fission in the brain^32^. We first examined the expression levels of Drp1 and phosphorylated Drp1 (pDrp1, at Ser^616^) in the hippocampus of HFD-fed, leptin-deficient (ob/ob), and leptin receptor-deficient (db/db) mice (Supplementary Fig. S5A–E). Although all three of these mouse models revealed insulin resistance and increased body weights, a significant reduction in brain weights was observed in ob/ob and db/db mice but not in the HFD-fed mice (Supplementary Table S1). In addition, hippocampal Drp1 expression in the mitochondrial fraction was significantly increased in the ob/ob and db/db mice but not in the HFD-fed WT mice (Supplementary Fig. S6A–C). Similarly, the levels of pDrp1 expression also increased in the hippocampi of the ob/ob and db/db mice (Supplementary Fig. S6D, E). These results showed that predominant mitochondrial fission occurs in obese or diabetic mice, which could cause mitochondrial dysfunction in hippocampal neurons.

**Fig. 4:**
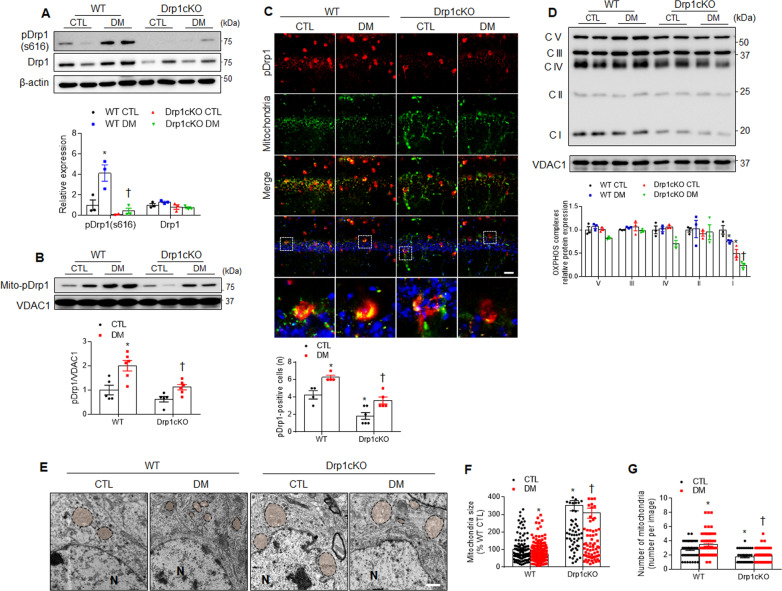
Effects of Drp1 deletion on mitochondrial dysfunction in CA1 hippocampal neurons in diabetic mice. **A** Western blot analysis of pDrp1 and Drp1 expression, using β-actin as a reference protein (*n* = 3–4 each group). **B** Western blot showing mitochondrial (mito-) pDrp1 expression from mitochondrial extract of the hippocampus. Densitometry values for pDrp1 were normalized against those for mitochondrial marker VDAC1 (*n* = 3–4 each group). **C** Representative images of immunofluorescence staining against pDrp1 (red) and mitochondria (green) in the hippocampal CA1 region. Nuclei were counterstained with DAPI (blue) (*n* = 3 mice per group). The pDrp1-positive cells in the hippocampus were measured and presented as the fold change. Scale bar = 20 µm. **D** Western blots and quantification of OXPHOS in the hippocampus (*n* = 3–4 mice per group). Densitometry values for OXPHOS were normalized against those for mitochondrial marker VDAC1 (*n* = 3–4 each group). **E** Representative electron microscopy images showing mitochondrial morphology in CA1 hippocampal neurons (*n* = 3 mice per group). N nucleus. Magnification, 10,000×. Scale bar = 1 µm. **F**, **G** Measurements of mitochondrial size (**F**) and mitochondrial numbers (**G**) in the CA1 pyramidal neuron layer or the stratum pyramidale. The indicated *p* values represent a two-way ANOVA followed by Tukey’s post hoc test. Data are shown as the mean ± SEM. *p < 0.05 vs WT CTL; ^†^*p* < 0.05 vs WT DM.

Notably, we found that hippocampal pDrp1 expression in total lysates was significantly increased in the WT DM mice compared with the WT CTL mice, whereas no changes were observed in the *Drp1cKO* mice (Fig. 4A). In addition, because the phosphorylation of Drp1 at Ser^616^ results in mitochondrial translocation and the promotion of mitochondrial fission, we measured the levels of pDrp1 in the mitochondria (Fig. 4B). Immunofluorescence analyses also showed an increase in the levels of mitochondrial pDrp1 expression in the WT DM mice, which decreased following Drp1 deletion (Fig. 4C). Although the expression level of mitochondrial fission factor was also reduced in the *Drp1cKO* mice compared with the WT mice, its expression was not affected by the induction of diabetes (Supplementary Fig. S7A). In contrast to the changes observed in Drp1 expression, no changes in mitochondrial fusion proteins, including mitofusin2 and optic atrophy1, were observed in any of the mice examined (Supplementary Fig. S7B, C). When oxidative phosphorylation (OXPHOS) was examined, the WT DM and *Drp1cKO* mice were found to have lower mitochondrial complex I expression than the WT CTL mice. Especially, the expression of mitochondrial complex I in the *Drp1cKO* DM mice was markedly decreased in the hippocampus of compared with the WT DM mice (Fig. 4D). Taken together, these results suggested that Drp1-regulated mitochondrial balance could play a critical role in neuroprotection against diabetes-induced synaptic injury. Electron microscopic findings showed that many small mitochondria are observed to be scattered around the pyramidal neuron nuclei located in the layer of the stratum pyramidale of the hippocampus of the WT DM mice compared with the WT CTL mice (Fig. 4E). In contrast, large mitochondria were observed in the *Drp1cKO* CTL and DM mice compared with the WT group. Mitochondrial size and number were significantly increased and decreased in the *Drp1cKO* DM mice compared with the WT DM mice, respectively (Fig. 4F, G). These findings indicated that as well as small mitochondria by Drp1, Drp1 deletion-induced large mitochondria could also cause mitochondrial dysfunction in the diabetic brain.

### Effects of Drp1 deletion on mitochondrial autophagy in the hippocampal CA1 region of HFD/STZ-induced diabetic mice

**Fig. 5:**
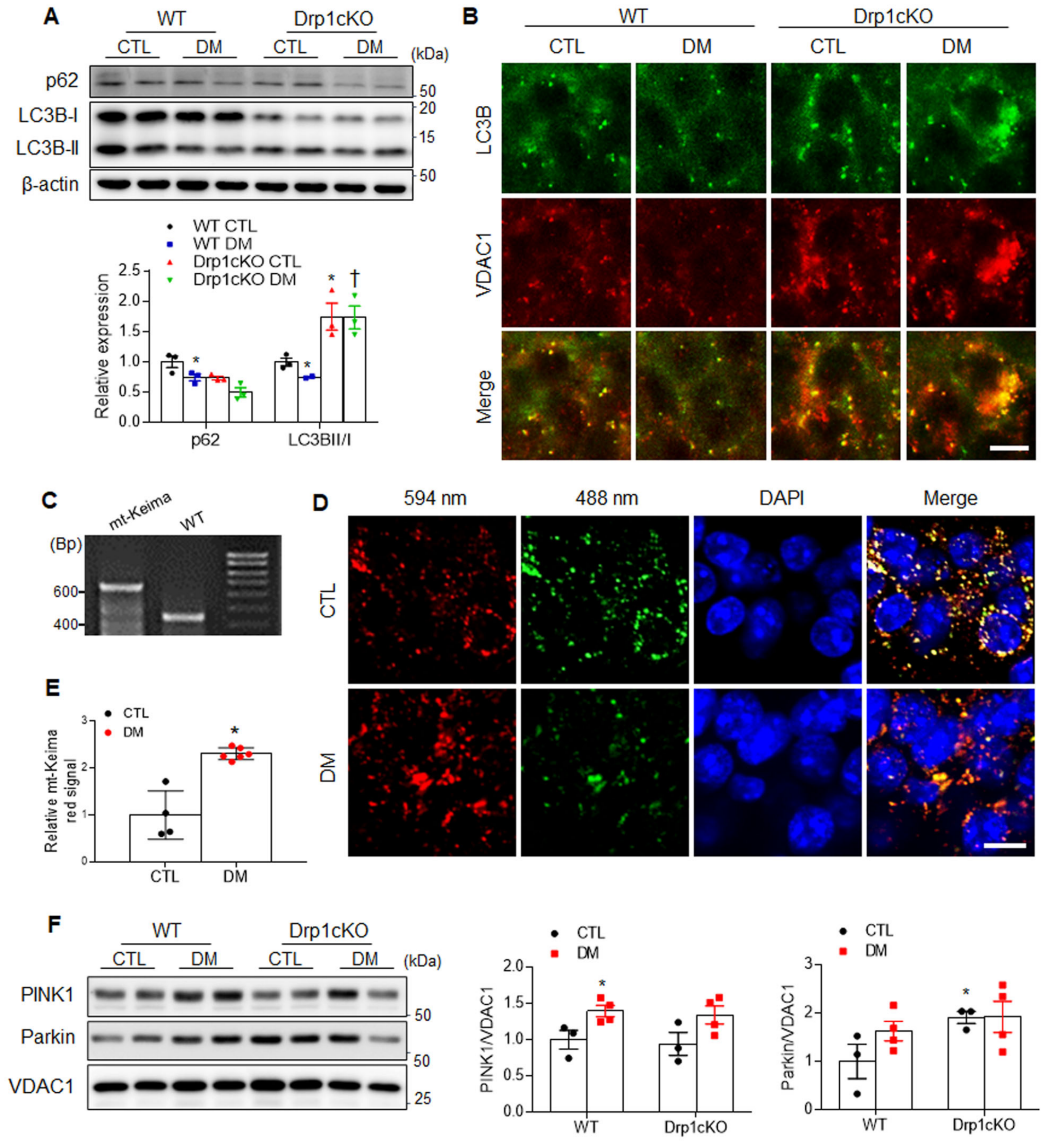
Effect of Drp1 deletion on mitochondrial autophagy in CA1 hippocampal neurons of diabetic mice. **A** Western blot analysis results showing the hippocampal p62 and LC3BI/II expression levels using β-actin as a loading control (*n* = 3–4 mice per group). **B** Representative double immunostaining of VDAC1 (red) and LC3B (green). Scale bar = 50 µm. **C** PCR analysis from the mt-Keima (Tg^+/+^) and WT (Tg^–/–^) mice was performed. **D** Representative confocal images showing mitophagy (Red pixel). Nuclei were counterstained with DAPI (blue). Scale bar = 10 µm. **E** Relative mt-Keima red signal in the hippocampal CA1 regions of CTL and DM mt-Keima (Tg^+/+^) mice. Values are normalized to CTL levels of mitophagy (*p* = 0.0003). **F** Western blot analysis results showing the hippocampal PINK1 and Parkin expression levels from mitochondrial extracts using mitochondrial marker VDAC1 as a loading control (*n* = 3–4 mice per group). The indicated *p* values represent a two-way ANOVA followed by Tukey’s post hoc test. Data are shown as the mean ± SEM. *p < 0.05 vs WT CTL; ^†^*p* < 0.05 vs WT DM.

Dysfunctional autophagy is closely associated with metabolic disorders, such as obesity and diabetes^33^. Given that increment on light-chain protein B (LC3B)-II expression could correlate with increased levels of autophagic vesicles or autophagosomes^34^, we evaluated changes in the expression levels of autophagy-related proteins, including p62 and the microtubule-associated 1 LC3B, in the hippocampus of HFD-fed, ob/ob, and db/db mice (Supplementary Fig. S8). We found that both p62 levels and the ratio of LC3BII/I were decreased in the hippocampus of various models of diabetic mice compared with control mice (Supplementary Fig. S8A–C). Consistent with other diabetic mouse models, p62 levels and the LC3BII/I ratio were lower in the WT DM mice than in the WT CTL mice (Fig. 5A). However, Drp1 deletion adversely increased the LCBII/I ratio in both CTL and DM mice. Immunofluorescence also revealed that high-intensity, LC3B-positive cells co-localized with VDAC1-positive mitochondria in CA1 hippocampal neurons in the *Drp1cKO* mice compared with the WT mice (Fig. 5B). These findings indicated that autophagy activation can be attenuated by metabolic disturbance and that this response can be paradoxically triggered by Drp1 loss.

In addition to the normal autophagic process, mitophagy is another mechanism that is necessary for the maintenance of mitochondrial quality^35^. We first assessed mitophagy using a transgenic mouse that expresses the mt-Keima (Fig. 5C). Mt-Keima, which is a mitochondria-associated, pH-sensitive, fluorescent protein, has previously been used as a convenient and accurate method for measuring mitochondrial autophagy^35^. As shown in Fig. 5D, E, the CA1 hippocampal neurons of 6-month-old diabetic mt-Keima mice showed increased levels of mt-Keima red signal compared with CTL mt-Keima mice (*p* = 0.0003). To further determine the involvement of mitophagy in diabetic hippocampal injury, we evaluated changes in the expression levels of mitophagy markers phosphatase and tensin homolog-induced putative kinase1 (PINK1) and parkin. As shown in Fig. 5F, HFD/STZ treatment induced increases in hippocampal PINK1 from mitochondrial extracts, but not in Parkin. In particular, Drp1 deletion increased hippocampal Parkin in *Drp1cKO* mice with or without HFD/STZ. These data indicated that imbalanced mitochondrial dynamics dominantly regulated by Drp1 could contribute to increased mitophagy under diabetic conditions.

### Effects of Drp1 deletion on mitochondrial autophagosome in the hippocampal neurons of HFD/STZ-induced diabetic mice

**Fig. 6:**
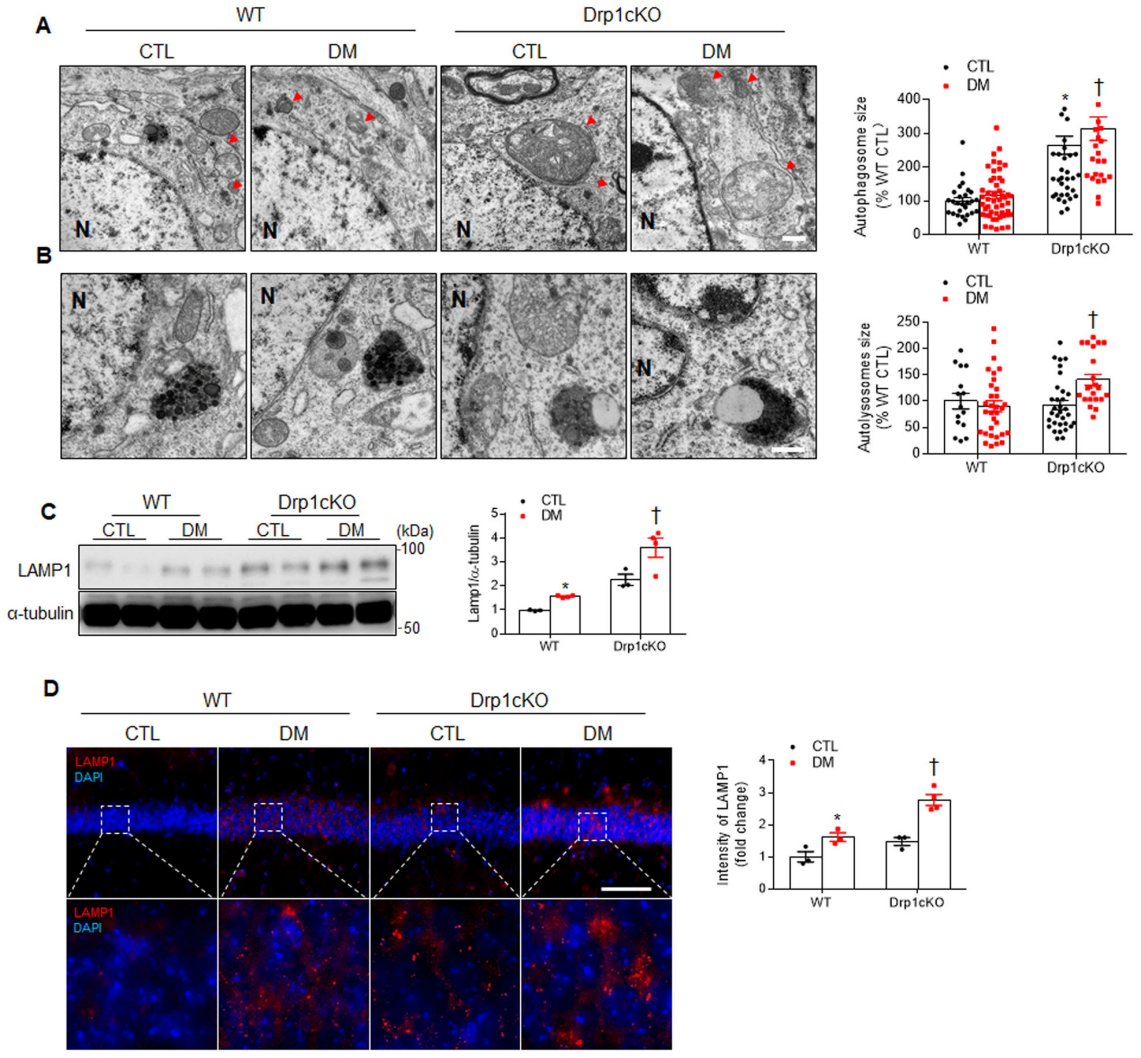
Effects of Drp1 deletion on mitochondrial autophagosome and lysosome in CA1 hippocampal neurons of diabetic mice. **A** Representative EM images showing autophagosome and **B** autolysosome sizes in CA1 hippocampal neurons from WT CTL, WT DM, *Drpc1KO* CTL, and *Drp1cKO* DM mice (*n* = 3 mice per group). Red arrow heads indicate autophagosomes containing small or large mitochondria. N nucleus. Magnification, 30,000×. Scale bar = 0.5 µm. **C** Western blot showing LAMP1 expression. **D** Representative staining of LAMP1 (red) in the hippocampal CA1 region. High-magnification images (in lower panels) from white box in upper panels. Nuclei were counterstained with DAPI (blue). The intensity of LAMP1 immunofluorescence in the hippocampus was measured and presented as the fold change. Scale bar = 50 µm. The indicated *p* values represent a two-way ANOVA followed by Tukey’s post hoc test. Data are shown as the mean ± SEM. *p < 0.05 vs WT CTL; ^†^*p* < 0.05 vs WT DM.

Large mitochondria are eliminated by autophagosomes^36^; therefore, we evaluated how autophagy is used to remove Drp1 loss-induced large mitochondria. We found autophagosomes containing small mitochondria in both the WT CTL and DM mice, whereas many atypical autophagosomes with large mitochondria were observed in the *Drp1cKO* mice (Fig. 6A). Dysregulated mitochondria are sequestered in double-membrane autophagosomes and delivered to lysosomes for degradation^37^. As shown in Fig. 6B, we only found large-sized double-membrane autophagosomes in the *Drp1cKO* DM mice. Finally, we examined whether Drp1 deletion affects lysosomal activity in the diabetic hippocampus. Diabetes-induced lysosome-associated membrane glycoprotein 1 (LAMP1) expression was predominantly increased by Drp1 deletion (Fig. 6C, D). Taken together, these findings suggested that Drp1 deletion could promote mitochondrial lysosomal pathway in the diabetic brain.

## Discussion

In the current study, we report that Drp1 loss-mediated mitochondrial abnormalities promote synaptic damage in HFD/STZ-induced diabetic brain, which is especially susceptible to oxidative stress and neuroinflammation. In particular, diabetic Drp1-deleted mice were more susceptible to synaptic injury and this diabetic *Drp1cKO* mice resulted in an increase in oxidative stress, neuroinflammation, synaptic damage, and mitochondrial lysosome in the hippocampus compared to diabetic WT mice. Notably, Drp1 deletion-induced large mitochondria within hippocampal neurons were engulfed by atypical large autophagosomes. Therefore, we suggest that lack of Drp1 in mouse hippocampus could exacerbate synaptic damage, neuroinflammation, and oxidative stress, which were dramatically aggravated by HFD/STZ-induced diabetes.

The brain is highly enriched with mitochondria and is heavily dependent on OXPHOS to generate energy for synaptic activity. In particular, changes in mitochondrial morphology under diabetic conditions have been shown to result in impaired respiratory enzyme function and dysfunctional mitochondrial bioenergetics^15,16,38^. HFD-fed obese and diabetic db/db mice have been shown to display memory deficits and an increase in dendritic spine loss in the prefrontal cortex and hippocampus^29,39^. Type 1 diabetes, combined with mitochondrial dysfunction in an AD mouse model, was previously reported to have synaptic defects^40^. Oettinghaus et al. reported that genetic Drp1 ablation in adult forebrain neurons induced hippocampal atrophy, without overt neurodegeneration^41^. Some studies, however, have reported that Drp1 deficiency resulted in enlarged mitochondrial morphology, leading to neurodegeneration, based on various experimental conditions^12,42–44^. In the hippocampus of our WT DM mouse model, we observed dendritic spine loss, and tau phosphorylation, which were also observed in the *Drp1cKO* mice, either with or without diabetic model induction. Moreover, the *Drp1cKO* DM mice exhibited more dendritic synapse loss than the WT DM mice. Drp1 loss-induced large mitochondria were observed near the nuclei of hippocampal neurons. However, few large mitochondria were observed in presynaptic areas within that stratum radiatum. Based on these findings, we hypothesized that the inhibition of mitochondrial movement down narrow, distal axons in the *Drp1cKO* mice could result in synaptic damage to the distal axons. Therefore, our findings suggested that Drp1 loss-induced large mitochondria could limit synaptic transmission and axonal bioenergetics in the diabetic brain.

Type 2 diabetes is characterized by mitochondrial dysfunction, high ROS synthesis, oxidative stress, and neuroinflammation^45^. Some studies have suggested that the upregulation of neuronal HO-1 may be associated with protective oxidative stress, improving cognitive dysfunction in elderly rats^46,47^. In contrast, HO-1 activation can increase tissue damage, depending on the intensity and duration of activation^48^. Our findings showed that the *Drp1cKO* DM mice exhibited an elevation in the levels of Nrf2 and HO-1 compared with the WT DM mice, suggesting that a stronger oxidative stress response may be triggered by Drp1 loss-induced mitochondrial dysfunction and synaptic injury. Furthermore, neuroinflammation can alter synaptic morphology and promote neurodegeneration^49,50^. Consistent with our findings, neurons damaged by ROS have been shown to exhibit changes in oxidative stress, neuroinflammation, and glial activation in ob/ob mice^51,52^. Taken together, these findings suggested that the inhibition of mitochondrial fission may promote oxidative stress and neuroinflammation in the diabetic brain.

Several findings have indicated that diabetes and neurodegenerative diseases, including Parkinson’s disease and AD, are correlated with the upregulation of hippocampal Drp1^19,53–55^. In addition, an increase in mitochondrial fission has been associated with hyperglycemia in the dorsal root ganglion neurons of diabetic mice^16^. Consistent with the increased Drp1 expression reported in ob/ob and db/db mice, an increase in the expression levels of Drp1 and pDrp1 (at Ser^616^) was also reported in the hippocampus of db/db mice^13^. Consistent with these findings, we also found decreased levels of Ser^616^ pDrp1 in the *Drp1cKO* DM mice, which was associated with enlarged mitochondrial morphology, compared with the WT DM mice^41,56^. The mitochondrial swelling induced by Drp1 loss may, therefore, be associated with an increase in neurodegeneration^57^. In the diabetic hippocampus, Drp1 expression increased to meet the demand for neuronal ATP energy, which is not static. Therefore, the *Drp1cKO* mice demonstrated serious synaptic damage in the diabetic brain, due to the inhibition of mitochondrial fission. Taken together, our results indicated that neuronal Drp1 deletion may accelerate neurodegeneration in the diabetic brain, due to dysregulated mitochondrial function.

LC3B and p62 levels were also decreased under diabetic conditions, due to the dysregulation of cell autophagy, which was mediated by the increased phosphorylation of signal transducer and activator of transcription 3^58^. Our data also showed decreased hippocampal LC3BII and p62 expression levels in HFD-fed, ob/ob, and db/db mice compared with control mice. However, we found that the ratio of LC3BII/I increased in the *Drp1cKO* mice compared with the WT DM mice, and many LC3B-positive puncta within the mitochondria were observed in the *Drp1cKO* mice. LC3B expression is an autophagosome marker, and the accumulation of LC3B-positive puncta has been associated with impaired autophagosome function^59^. We proposed that an increased LC3B expression can either indicate an increase in autophagosome formation or impairment in its degradation^60^. In addition to LC3 turnover, the level of p62 proteins can be used to monitor autophagic flux^61^. Autophagic activation correlates with a decreased p62 level. Consistent with the evidence that DM causes reduced p62 level, Drp1cKO mice also have reduction of p62 protein. Thus, these findings suggested that Drp1 deletion may play an important role in impairing the autophagic process.

Some studies have shown that Drp1 is not necessary for mitophagy in yeast and mammals^10,62–64^. Yamashita et al. demonstrated that autophagosome biogenesis is initiated on the mitochondrial tubules and induces the division of the mitochondrial fragment, along with the expansion of the isolation membrane, in *Drp1* KO HeLa cells^63^. In contrast, one study has suggested that a significantly enlarged autophagosome could affect the clearance of damaged, swollen mitochondria^65^. Reduced LC3B expression has been reported in patients with diabetes and may be related to adaptive mitophagy^66^. Previous studies have reported that PINK1-Parkin-mediated mitophagy is activated in ischemia-reperfusion-induced cerebral or renal injury, which can protect against renal damage^67,68^. Under diabetic conditions, our findings also showed that diabetic mt-Keima mice demonstrated an increase in mitophagy induction compared with non-diabetic mice. In addition, we found that elevated PINK1 protein level indicates protective effects of diabetic hippocampal injury, whereas diabetic Drp1cKO mice also promote mitophagy. This indicates an increase in the ability of hippocampal neurons to clear damaged mitochondria. In the present study, we showed that hippocampal LAMP1 expression was significantly increased in the *Drp1cKO* DM mice, with large autophagosomes, compared to the WT DM mice. These findings indicated that increased autophagosome and autolysosome formation could be involved in the removal of large mitochondria caused by neuronal Drp1 loss.

In conclusion, our study provides the mechanisms for the adverse effects of Drp1 loss-mediated mitochondrial dynamics on synaptic damage caused by HFD/STZ-induced diabetes. The diabetic *Drp1cKO* mice displayed more neurodegeneration including synaptic damage, oxidative stress, and neuroinflammation compared to diabetic WT mice. Using EM analysis, we clearly demonstrated alternation in mitochondrial structure and clearance of defective mitochondria in the diabetes-affected hippocampus. Notably, this study showed that Drp1 loss-induced large mitochondria within hippocampal neurons are engulfed by large autophagosomes. Therefore, we suggest that neuronal mitochondrial dynamics mediated by Drp1 may be critical for synaptic formation and cognitive function.

## Supplementary information

Supplementary figures and tables
