# Correction: Ablation of dynamin-related protein 1 promotes diabetes-induced synaptic injury in the hippocampus

**DOI:** 10.1038/s41419-021-03841-2

**Published:** 2021-06-01

**Authors:** Gyeongah Park, Jong Youl Lee, Hye Min Han, Hyeong Seok An, Zhen Jin, Eun Ae Jeong, Kyung Eun Kim, Hyun Joo Shin, Jaewoong Lee, Dawon Kang, Hyun Joon Kim, Yong Chul Bae, Gu Seob Roh

**Affiliations:** 1 Department of Anatomy and Convergence Medical Science, Institute of Health Sciences, College of Medicine, Gyeongsang National University, Jinju, Gyeongnam 52727 Republic of Korea; 2 Bio Anti-Aging Medical Research Center, College of Medicine, Gyeongsang National University, Jinju, Gyeongnam 52727 Republic of Korea; 3 Department of Anatomy and Neurobiology, University of Tennessee Health Science Center, Memphis, TN 38163 USA; 4 Department of Anatomy and Neurobiology, School of Dentistry, Kyungpook National University, Daegu, 41944 South Korea; 5 Department of Physiology, College of Medicine, Institute of Health Sciences, Gyeongsang National University, Jinju, Gyeongnam 52727 Republic of Korea

**Keywords:** Neurodegeneration, Neurodegeneration, Diabetes complications

Correction to: *Cell Death and Disease*

10.1038/s41419-021-03723-7 published online 5 May 2021

The original version of this article unfortunately contained two mistakes. In the Materials and methods and the Acknowledgement section it should read “Dr. Jeanho Yun (Dong-A University, Busan, South Korea)”. The authors apologize for the mistake. The original article has been corrected.

