# Supplementary material for: Ablation of dynamin-related protein 1 promotes diabetes-induced synaptic injury in the hippocampus
Source: Cell Death Dis. 2021 May 5;12(5):445. doi: 10.1038/s41419-021-03723-7 (PMC8099876; doi:10.1038/s41419-021-03723-7)
Supplement: Supplementary file 1 — Supplementary figures and tables [file 41419_2021_3723_MOESM1_ESM.docx]

**Ablation of dynamin-related protein 1 promotes diabetes-induced synaptic injury in the hippocampus**

Gyeongah Park^1,2,3^, Jong Youl Lee^1,2^, Hye Min Han^4^, Hyeong Seok An^1,2^, Zhen Jin^1,2,3^, Eun Ae Jeong^1,2^, Kyung Eun Kim^1,2^, Hyun Joo Shin^1,2^, Jaewoong Lee^1,2^, Dawon Kang^2,5^, Hyun Joon Kim^1,2^, Yong Chul Bae^4^, Gu Seob Roh^1,2*^

^1^Department of Anatomy and Convergence Medical Science, Institute of Health Sciences, College of Medicine, Gyeongsang National University, Jinju, Gyeongnam 52727, Republic of Korea

^2^Bio Anti-Aging Medical Research Center, College of Medicine, Gyeongsang National University, Jinju, Gyeongnam 52727, Republic of Korea

^3^Department of Anatomy and Neurobiology, University of Tennessee Health Science Center, Memphis, TN 38163, USA

^4^Department of Anatomy and Neurobiology, School of Dentistry, Kyungpook National University, Daegu 41944, South Korea

^5^Department of Physiology, College of Medicine, Institute of Health Sciences, Gyeongsang National University, Jinju, Gyeongnam 52727, Republic of Korea

**
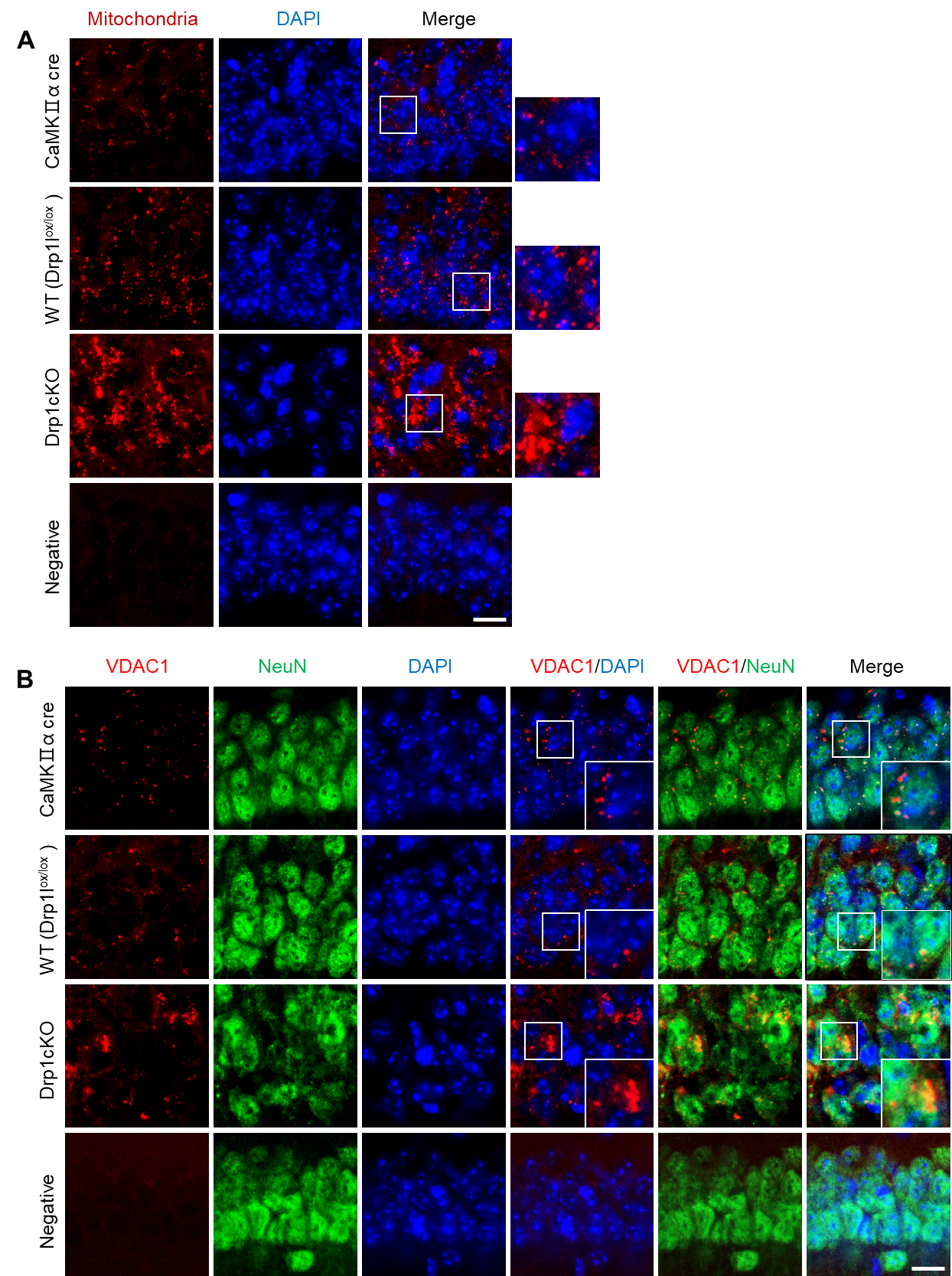
**

**Supplementary Figure 1. Effects of Drp1 deletion on perinuclear mitochondria morphology in WT, CaMKⅡα cre, and *Drp1cKO* mice.** (A) Representative immunofluorescence staining against mitochondria (red) and DAPI (blue) in the hippocampal CA1 region. High-magnification images (in right panels) from white box in left panels. Scale bar = 10 µm. (B) Representative staining of VDAC1 (red) and NeuN (green) in the hippocampal CA1 region. Nuclei were counterstained with DAPI (blue). High-magnification images from small white box. Scale bar = 10 µm.

**
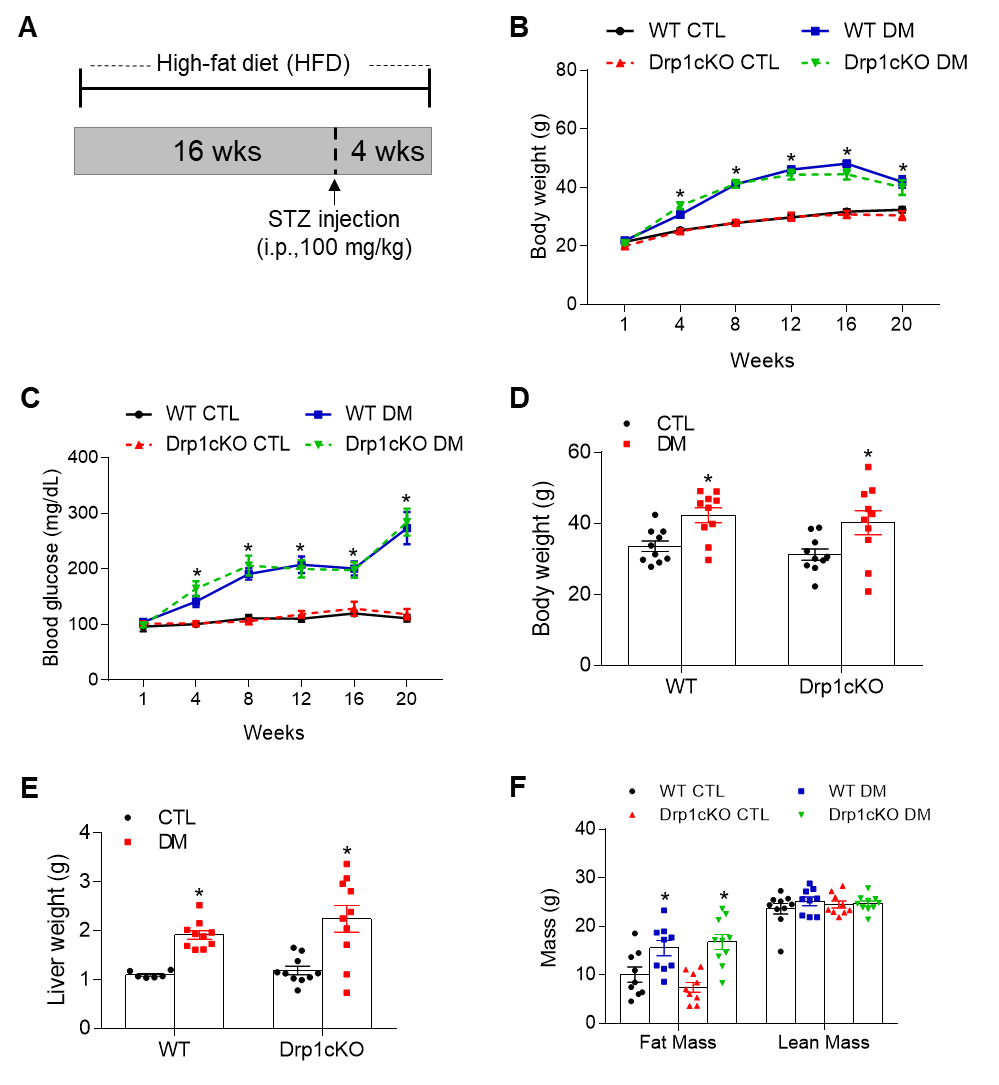
**

**Supplementary Figure 2. HFD/STZ treatment-induced diabetic phenotypes in WT and *Drp1cKO* mice.** (A) Schematic representation of the experimental protocol. (B) Body weight and (C) fasting blood glucose levels of mice after 20 weeks (n = 12 mice per group). (D) Body weight; (E) liver weight; (F) fat mass and lean mass (n = 9–10 mice per group). Data are shown as the mean ± SEM. The indicated *p*-values represent a two-way ANOVA followed by Tukey’s post-hoc test. **p*<0.05 vs WT CTL.


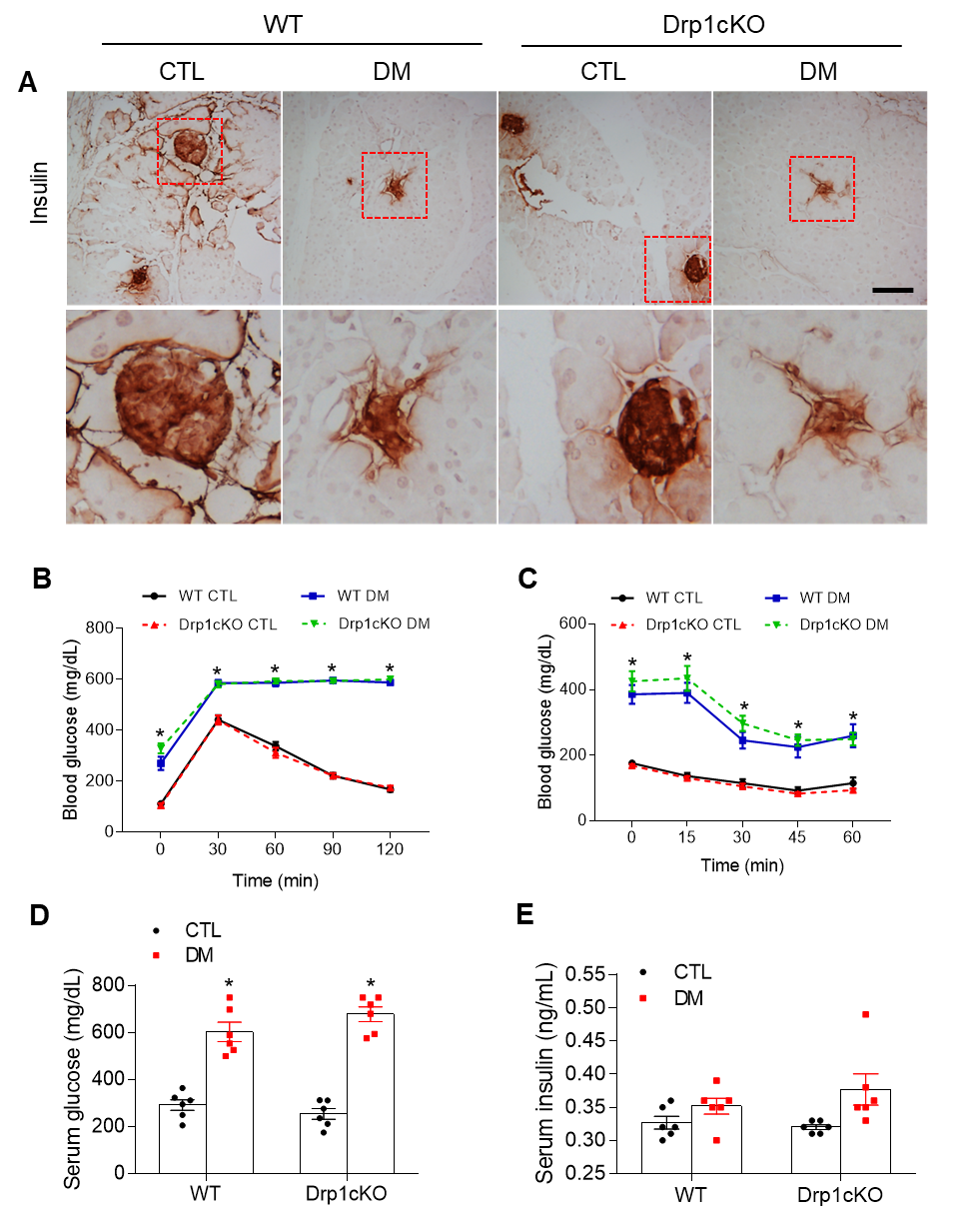


**Supplementary Figure 3. HFD/STZ-induced diabetic mice exhibit insulin resistance.** (A) Representative microphotographs, showing immunostained insulin in pancreatic islets. High- magnification images (in lower panels) from red box in upper panels. Scale bar = 100 µm. (B) Glucose tolerance test; (C) Insulin tolerance test (n=10 mice per group). (D) Serum glucose levels; (E) Serum insulin levels, using ELISA (n = 6 mice per group). Data are shown as the mean ± SEM. The indicated *p*-values represent a two-way ANOVA followed by Tukey’s post-hoc test. **p*<0.05 vs WT CTL.

**
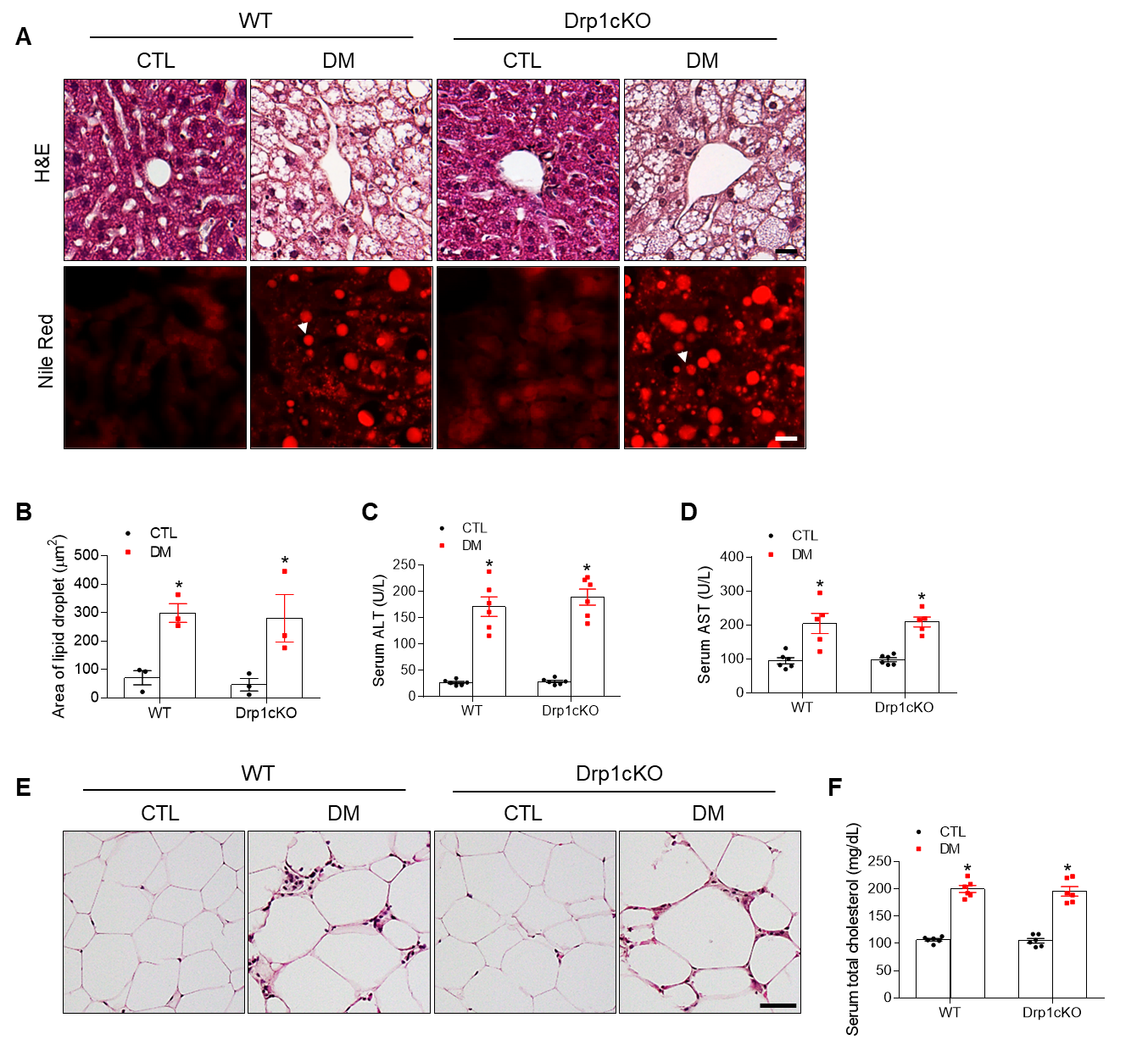
Supplementary Figure 4. HFD/STZ-induced diabetic mice exhibit hepatic steatosis and macrophage infiltration.** (A) Representative microphotographs of H&E and Nile red staining in liver sections. Scale bar = 20 µm. Arrow heads indicate Nile red-positive lipid droplets. (B) Percentages of Nile red-positive areas (n = 3–4 mice per group). (C) Alanine aminotransferase (ALT) levels and (D) aspartate aminotransferase (AST) levels (n = 6 mice per group). (E) Representative images of H&E staining in epididymal fat pad sections. Scale bar = 50 µm. (F) Total cholesterol (n = 6 mice per group). Data are shown as the mean ± SEM. The indicated *p*-values represent a two-way ANOVA followed by Tukey’s post-hoc test. **p*<0.05 vs WT CTL.

**
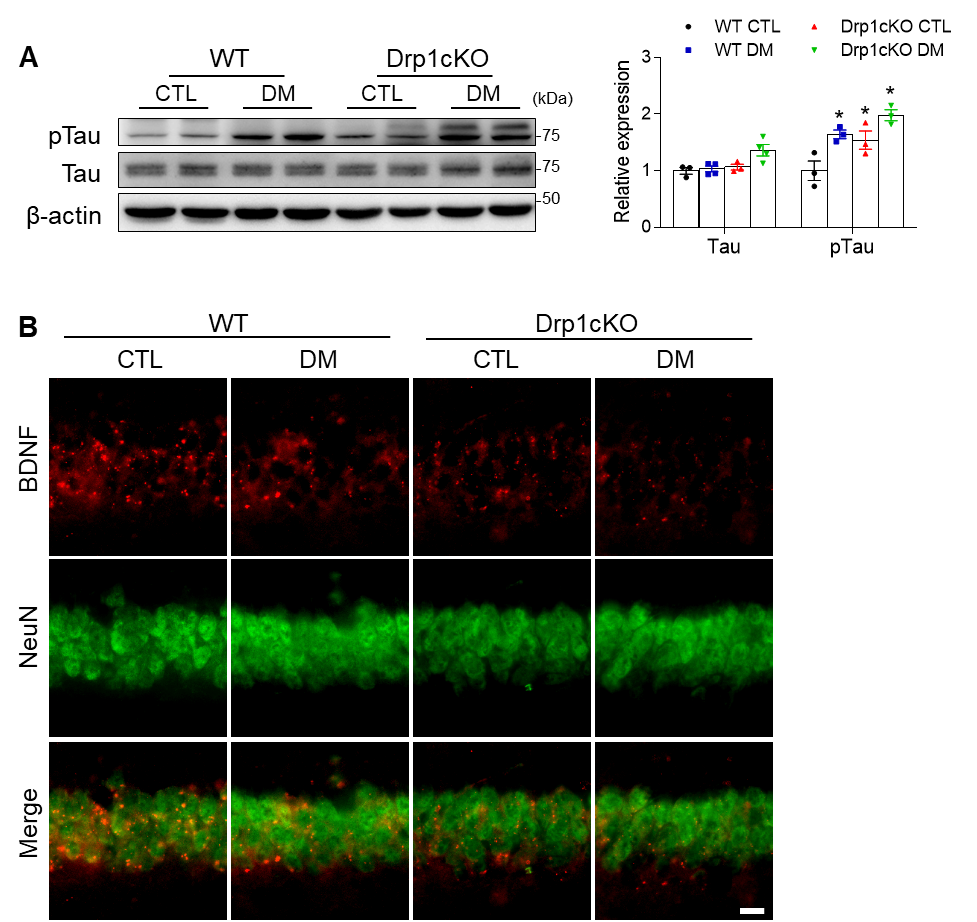
**

**Supplementary Figure 5. Effects of HFD/STZ treatment on hippocampal Tau, BDNF expression, and memory deficits in WT and *Drp1cKO* mice.** (A) Western blot showing phosphorylated Tau and total Tau protein expression in the hippocampus from each group (n = 3–4 mice per group). (B) Representative double-immunofluorescence staining against BDNF (red) and NeuN (green) in the hippocampus. Scale bar = 10 µm. The indicated *p*-values represent a two-way ANOVA followed by Tukey’s post-hoc test. **p*<0.05 vs WT CTL.

**
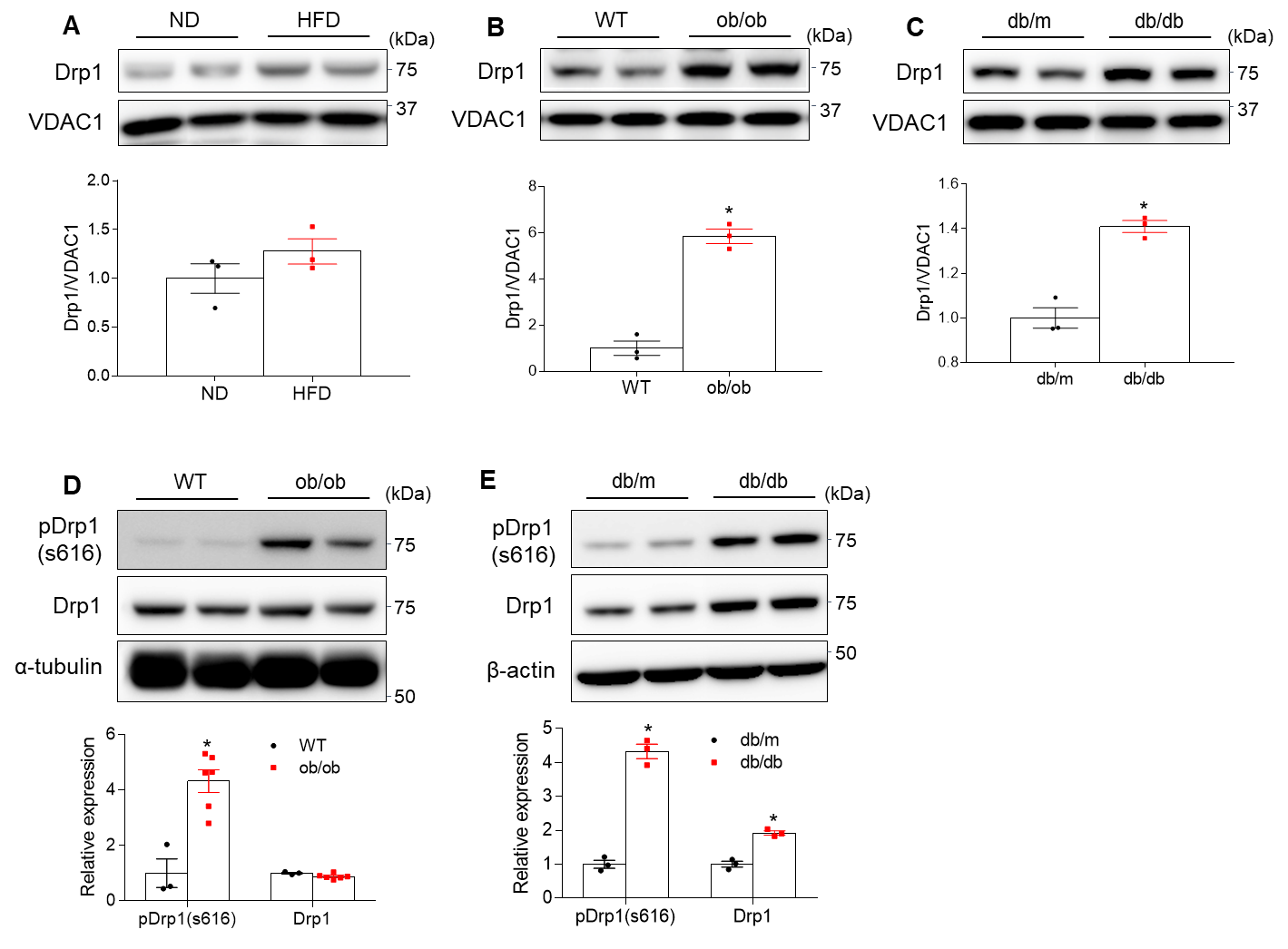
**

**Supplementary Figure 6. Increased hippocampal Drp1 expression in HFD-fed, ob/ob, and db/db mice.** Western blot analysis of Drp1 protein expression in (A) HFD-fed, (B) ob/ob, and (C) db/db mice, using VDAC1 as a reference protein (n = 3–4 mice per group). Western blot analysis showing pDrp1 expression in (D) ob/ob and (E) db/db mice. α-tubulin and β-actin were used as loading controls, respectively. The indicated *p-*values represent unpaired *t*-test in **A**, **B**, and **C** or two-way ANOVA in **D** and **E** followed by Tukey’s post-hoc test. **p*<0.05 vs WT (db/m) mice and shown as the mean ± SEM.

**
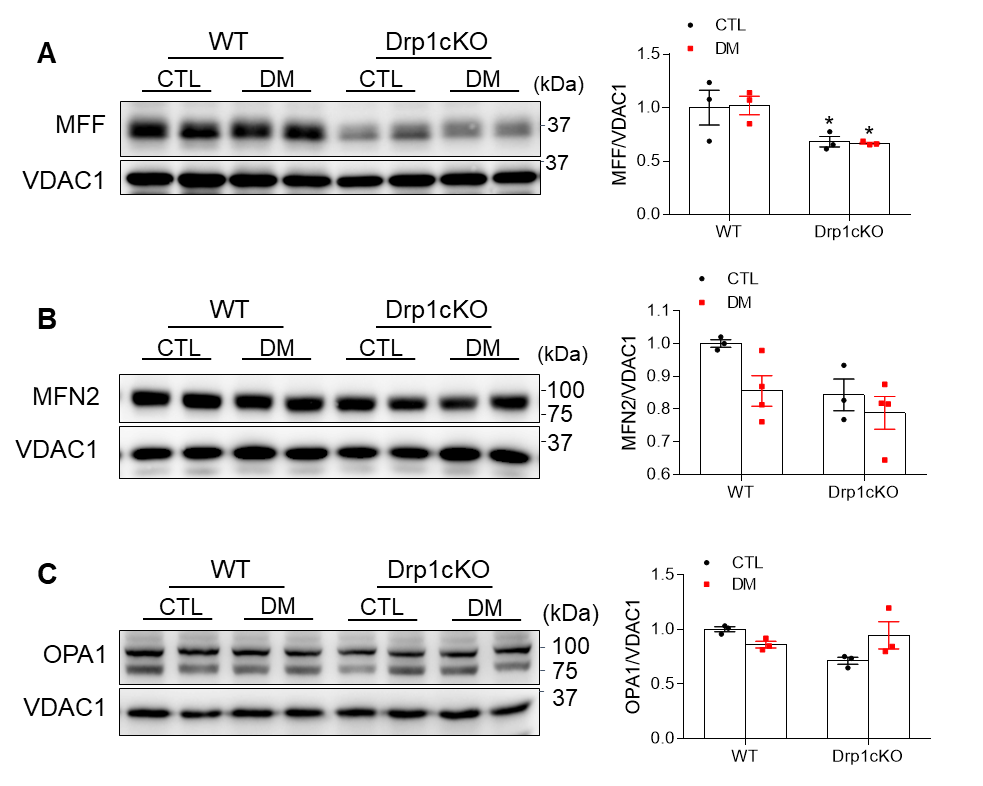
**

**Supplementary Figure 7. Effect of Drp1 deletion on mitochondria fission protein and OXPHOS activity in the hippocampus of HFD/STZ-induced diabetic mice.** Western blot analysis showing (A) MFF, (B) MFN2, and (C) OPA1 expression levels in the hippocampus, using VDAC1 as a loading control (n = 3–4 mice per group). Data are shown as the mean ± SEM. The indicated *p*-values represent a two-way ANOVA followed by Tukey’s post-hoc test. **p*<0.05 vs WT CTL.

**
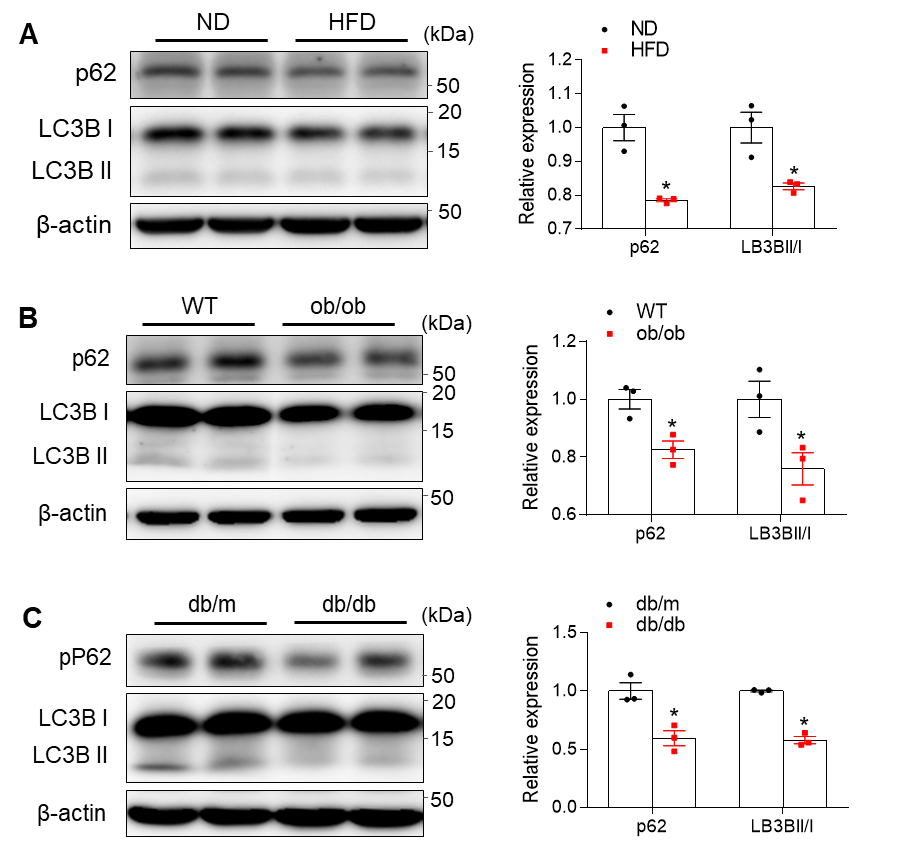
**

**Supplementary Figure 8. Effects of mitochondrial autophagy in HFD-fed, ob/ob, and db/db mice.** Western blot analysis results showing the hippocampal p62 and LC3B expression levels in (A) HFD-fed, (B) ob/ob, and (C) db/db mice, using β-actin as a loading control (n = 3–4 mice per group). Data are shown as the mean ± SEM. The indicated *p-*values represent unpaired *t*-test. **p*<0.05 vs ND or WT (db/m) mice.

**Supplementary Table 1. Changes in body and brain weights in HFD-fed, ob/ob, and db/db mice**

|  | ND | HFD | WT | ob/ob | db/m | db/db |
| --- | --- | --- | --- | --- | --- | --- |
| Body weight (g) | 33.92 ± 0.80 | 47.86 ± 1.10* | 29.60 ± 0.33 | 51.81 ± 5.55* | 34.97 ± 1.03 | 62.10 ± 2.74* |
| Brain weight (g) | 0.46 ± 0.01 | 0.48 ± 0.01 | 0.45 ± 0.01 | 0.37 ± 0.01* | 0.46 ± 0.01 | 0.37 ± 0.03^†^ |
| Brain/Body weight Ratio | 1.36 ± 0.03 | 1.01 ± 0.03* | 1.53 ± 0.02 | 0.78 ± 0.09* | 1.32 ± 0.04 | 0.60 ± 0.06* |
| Blood glucose (mg/dL) | 149.13 ± 7.19 | 177.29 ± 5.86^†^ | 152.60 ± 2.37 | 357.78 ± 68.80* | 167.17 ± 5.01 | 561.33 ± 24.98* |

The results are presented as the mean ± standard error of the mean (n = 6–10). Statistical analysis was performed using the Student’s t-test; **p*<0.01, ^†^*p*<0.05. ND, normal diet; HFD, high-fat diet; WT, wild-type

**Supplementary Table 2. List of primers**

| PCR | Primers | Sequences |
| --- | --- | --- |
| *Drp1 lox/lox* | D3  D6 | ACC AAA GTA AGG AAT AGC TGT TG  ATG CGC TGA TAA TAC TAT CAA CC |
| *CaMKIIα-Cre* | oIMR1084  oIMR1085  oIMR7338  oIMR7339 | GCG GTC TGG CAG TAA AAA CTA TC  GTG AAA CAG CAT TGC TGT CAC TT  CTA GGC CAC AGA ATT GAA AGA TCT  GTA GGT GGA AAT TCT AGC ATC C |
| *Mt-Keima* | H11-F1  5F  PR387 | AGT CTT TCC CTT GCC TCT GC  ACA ACC GCG ACT ACA CCA  GTG GGA CTG CTT TTT CCA GA |

**Supplementary Table 3. List of primary and secondary antibodies**

| **Antibody** | **Company** | **Catalog No** | **Dilution(s)** | | **Applications** | | | **Source** |
| --- | --- | --- | --- | --- | --- | --- | --- | --- |
| α-tubulin | Sigma-Aldrich | T5168 | 1:1000 | WB | | Mouse | | |
| ꞵ-actin | Sigma-Aldrich | A5441 | 1:1000 | WB | | Mouse | | |
| BDNF | Santacruz | Sc546 | 1:200 | IF | | Rabbit | | |
| Drp1 | Biosciences | BD611163 | 1:1000 | WB, IF | | Mouse | | |
| GAD65 | Abcam | Ab26113 | 1:500 | IF | | Mouse | | |
| GFAP | Sigma-Aldrich | G3593 | 1:250 | IF | | Mouse | | |
| HO-1 | Stressgen | SPA-895 | 1:1,000, 1:200 | WB, IF | | Rabbit | | |
| Iba-1 | Wako | 016-20001 | 1:250 | IF | | Rabbit | | |
| Insulin | Abcam | Ab7842 | 1:250 | IHC | | Guinea pig | | |
| LAMP1 | Abcam | Ab24170 | 1:200,1:1000 | WB, IF | | Rabbit | | |
| LC3B | Sigma-Aldrich | L7543 | 1:1000, 1:250 | WB, IF | | Rabbit | | |
| NeuN | Millipore | MAB377 | 1:1000 | IF | | Mouse | | |
| MFF | Abcam | Ab81127 | 1:1000 | WB | | Rabbit | | |
| MFN2 | Abcam | Ab56889 | 1:1000 | WB | | Mouse | | |
| Mitochondria | Abcam | Ab3298 | 1:200 | IF | | Mouse | | |
| NFκBp65 | Cell signaling | #6959 | 1:1000 | WB | | Mouse | | |
| Nrf2 | Abcam | Ab137550 | 1:1000 | WB | | Rabbit | | |
| OPA1 | Biosciences | BD612606 | 1:1000 | WB | | Mouse | | |
| OXPHOS | Abcam | Ab110413 | 1:5000 | WB | | Mouse | | |
| Parkin | Abcam | Ab15954 | 1:1000 | WB | | Rabbit | | |
| PINK1 | Santacruz | Sc33796 | 1:1000 | WB | | Rabbit | | |
| p62 | Sigma-Aldrich | P0067 | 1:1000 | WB | | Rabbit | | |
| p84 | Abcam | Ab487 | 1:3000 | WB | | Mouse | | |
| pDrp1 (Ser616) | Cell signaling | #3455 | 1:1000 | WB | | Rabbit | | |
| pTau (Ser396) | Thermo | 44-752G | 1:1000 | | WB | | Rabbit | |
| Tau | Santacruz | Sc1998 | 1:1000 | | WB | | Goat | |
| VDAC1 | Sigma-Aldrich | Ab15895 | 1:1000, 1:250 | | WB, IF | | Mouse | |

BDNF, brain-derived neurotrophic factor; Drp1, Dynamin-related protein; GFAP, Glial fibrillary acidic protein; HO-1, Heme oxygenase-1; Iba-1, Ionized calcium binding adaptor molecule; LAMP1, Lysosomal-associated membrane protein 1; LC3B, Microtubule-associated protein; NeuN, Neuronal nuclei; MFF, Mitochondrial fission factor; MFN2, Mitofusin 2; NFκBp65, nuclear factor-kappa-B p65; Nrf2, transcription factor NF-E2-related factor 2; OPA1, optic atrophy1; OXPHOS, Oxidative phosphorylation; Phosphatase and Tensin Homolog (PTEN)-induced putative kinase 1 (PINK1); pDrp1, Phosphorylated Drp1; pTau, Phosphorylated Tau; WB, western blot; IF, immunofluorescence; IHC, immunohistochemistry
